# Thermal adaptation and fatty acid profiles of bone marrow and muscles in mammals: Implications of a study of caribou (*Rangifer tarandus caribou*)

**DOI:** 10.1371/journal.pone.0268593

**Published:** 2022-12-01

**Authors:** Eugène Morin, Päivi Soppela, P. Yvan Chouinard

**Affiliations:** 1 Department of Anthropology, Trent University, Peterborough, Ontario, Canada; 2 Université de Bordeaux, Pessac Cedex, France; 3 Arctic Centre, University of Lapland, Rovaniemi, Finland; 4 Université Laval, Département des Sciences Animales, Pavillon Paul-Comtois, Québec, Canada; University of Veterinary Medicine Vienna: Veterinarmedizinische Universitat Wien, AUSTRIA

## Abstract

Mammals have evolved several physiological mechanisms to cope with changes in ambient temperature. Particularly critical among them is the process of keeping the membrane of cells in a fluid phase to prevent metabolic dysfunction. In this paper, we examine variation in the fatty acid composition of bone marrow and muscle tissues in the cold-adapted caribou (*Rangifer tarandus caribou*) to determine whether there are systematic differences in fatty acid profiles between anatomical regions that could potentially be explained by thermal adaptation as influenced by cell function, including hematopoiesis. Our results indicate that the bone marrow and muscle tissues from the appendicular skeleton are more unsaturated than the same tissues in the axial skeleton, a finding that is consistent with physiological adaptation of the appendicular regions to thermal challenges. Because mechanisms of thermal adaptation appear to be widely shared among terrestrial mammals, we suggest that the same patterns may prevail in other species, possibly including humans.

## Introduction

How mammals, including humans, adapt to changes in ambient temperature has long been a focus of intensive research in biology [1–5]. A critical challenge that all mammals must face is to maintain their high internal body temperature by conserving heat in cold environments and dissipating heat in hot environments and/or when the animal is conducting long, vigorous activity [6]. At the scale of individual cells, the problem concerns how the physical properties of the membrane and its composition can be modified in order to dynamically maintain a fluid (liquid-crystalline) phase under challenging thermal conditions [7]. At low temperatures, this means keeping the cell away from a gel phase, whereas at high temperature it implies avoiding the loss of bilayer integrity and membrane fusion [8]. Avoiding these changes in phase or structure is crucial because they can have deleterious effects on cell function [9], and at a larger scale, may result in stiff or loose tissues, with potentially adverse effects on locomotion, food procurement and the ability of an animal to respond swiftly in contexts of predation. In conditions of low ambient temperature, a common pattern seen in cells is desaturation, which consists in increased proportion of unsaturated fatty acids (FA) at the expense of saturated FA. At high ambient temperatures, these changes are commonly reversed [7, 9–11]. In this paper, we examine how the FA composition of skeletal marrow from different parts of the body varies—likely in response to thermal challenges [4]—in a species of cold climate, the caribou (*Rangifer tarandus caribou*). The FA composition in bone marrow is also compared with that of skeletal muscles to determine whether this tissue is similarly affected by exposure to ambient temperature.

Bone marrow adipocytes (BMA) are metabolically active cells that are currently intensively studied because they act as an energy reservoir, secrete important proteins (e.g., adiponectin, leptin) and influence local marrow processes, osteogenesis and systemic metabolism, among other functions [12–14]. In humans, the formation of BMA occurs at or shortly prior to birth with these cells gradually replacing hematopoietic tissue. This process follows a well established distal-to-proximal sequence: BMA first form in the terminal phalanges, then develop in the long bones, and later expand into the axial skeleton [15]. Within individual long bones, BMA first form at mid-shaft, then occur in the distal epiphysis, and ultimately invade the proximal epiphysis [16, 17]. Importantly, these developmental trends are not unique to humans and have been observed in many mammal species, including rodents, ungulates, and carnivores [e.g., 18, 19].

Recent studies have uncovered important molecular, functional and morphological differences between subtypes of BMA. In the epiphyseal regions of the long bones—loci generally associated with active hematopoiesis—BMA represent only approximately 45% of the cellular component in adults, which contrasts with their abundance in long bone cavities where they can constitute as much as 90% of the cellular tissue [20, 21]. The BMA found in hematopoietic regions also tend to be of smaller size, to be more dispersed, to develop later and to be more easily mobilized than the BMA found in the shaft cavities of the same bones. Moreover, BMA in epiphyseal and diaphyseal regions have been shown to differ in terms of patterns of regulation and gene expression [22, 23]. As a result of these differences, the adipocytes from the epiphyseal regions have been termed regulated BMA (rBMA, or “red marrow” in the older literature), whereas those found in the diaphyses or shafts of long bones are called constitutive BMA (cBMA, or “yellow marrow” in older publications) [22].

Research on terrestrial mammals has shown that the FA composition of BMA is influenced by several factors, including the anatomical locus under investigation as well as the age, diet, health, and gender of the individual [15, 24–26]. Because the FA composition of BMA appears to be influenced by tissue temperature, and indirectly by ambient temperature [4, 24, 27–29], comparing patterns in the axial skeleton with that from the more heterothermic limbs may yield important insights on the interactions between cell function (including hematopoiesis and fat storage) and thermal challenges. For instance, cBMA are known, to increase in unsaturation towards the extremities, and are thus kept fluider, in order to prevent stiffness at low temperatures [4, 24, 27–29]. Whether this pattern also applies to the adjacent muscles and to adipocytes in the epiphyseal regions is poorly known. This issue is important because, unlike cBMA, intramuscular fat in lean animals has a high content of structural lipids such as phospholipids and cholesterol [30–32], an observation that likely extends to the marrow in the epiphyseal regions given its hematopoietic functions. How the FA composition of muscle and bone marrow tissues varies within an animal and how these tissues are influenced by ambient temperature is understudied [but see 2, 33, 34]. The present study addresses this problem by investigating changes in the FA composition of these tissues across a large number of different anatomical sites in caribou. We also explore the implications of these variations for our understanding of the thermal adaptation of mammals.

## Materials and methods

### Sample collection

Whereas many previous studies of FA composition in wild mammals have investigated a single category of tissue (e.g., adipose tissue, bone marrow or a specific skeletal muscle) sampled across a large number (e.g., >10) of individuals at a small number of anatomical sites (e.g., 1–5 sites), here we used a different approach and focused on intra-individual variation. For this study, FA variation was examined at a large number of anatomical sites (*n* = 56 per individual) in caribou, with special attention being paid to a wide range of soft tissues, including backfat, skin, skeletal muscle, lungs and trachea, and bone marrow. This sampling strategy allows for a detailed picture of FA profile variation within a cold-adapted mammal.

The study was approved by the Animal Care Committee of Trent University. No animal was hunted for this study. The muscle and bone marrow tissues that we used were obtained from a commercial butcher and were part of meat products destined to restaurants and markets.

**Fig 1:**
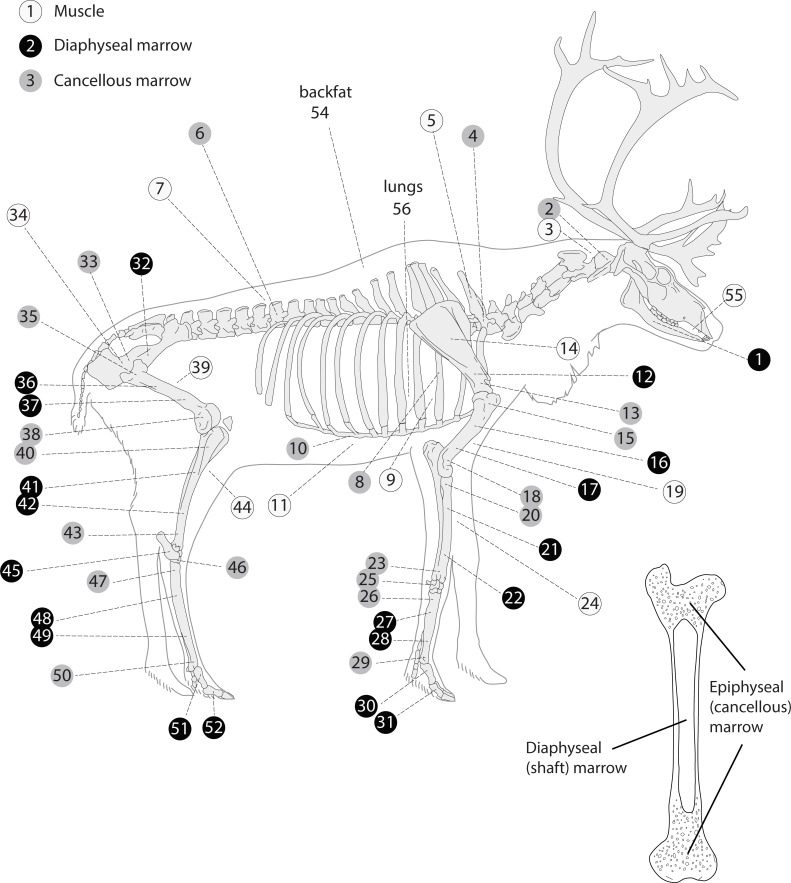
Anatomical sites sampled in caribou for this study. The numbers correspond to tissues listed in Table 1.

For this analysis, two already eviscerated female (≥6 year-old) caribou (*Rangifer tarandus caribou*) were sampled. Both free-ranging caribou were killed by hunters around February 7–10, 2007 in the Robert-Bourassa Reservoir (53°45’N, 77°00′W; female A) and the Caniapiscau region (53°00′N, 68°30′W; female B) in central Québec where average temperature is around -23°C in January and 13°C in July (Schefferville airport weather station). Carcass weights (excluding organs, visceras, brain and antlers) were 64 and 61 kg, respectively. The two animals were, based on visual inspection, apparently in good condition and showed no signs of pathology or being starved. Whether the animals were pregnant or not is unknown. As part of the meat aging process, carcasses were kept in a refrigeration facility (at ca 4°C) for about two weeks prior to processing by a commercial butcher. Fatty acid profiles were derived for a total of 112 samples (56 per caribou) collected from the skin, lungs and trachea, various muscles, and the bone marrow from most classes of skeletal elements (in the case of long bones, samples were taken from the epiphyseal and shaft portions of the bones). Fig 1 shows the anatomical location of the samples.

### Sample processing and FA analysis

How the marrow was obtained requires additional description. Because the FA analysis was performed with the aim of shedding light on human foraging decisions in prehistoric contexts—a topic that will be the focus of another publication—the marrow from the shaft cavity (diaphyseal marrow) of the long bones (humerus, radio-ulna, femur, tibia and metapodials) was extracted after breaking the bones using a pebble and a stone anvil as documented in a wide range of subsistence-based societies [35]. After the long bone shaft cavity was breached, a few grams of the exposed diaphyseal marrow was cut using a knife and then frozen in a plastic bag in a commercial freezer prior to FA analysis. One sample each was taken from the proximal and distal ends of the marrow plug. The cancellous marrow samples (marrow in the axial skeleton and girdles, carpals, tarsals, and epiphyseal regions of long bones; Fig 1) were obtained by crushing the specimen using the same stone-and-anvil method. This crushing yielded a product similar to bone meal or bone paste. This means that while the marrow extracted from the shaft cavity of the long bones was largely fat-like (or fatty) tissue and free of bone fragments, the crushed cancellous bone samples consisted of marrow-rich bone meal. As the bone tissue itself—that is excluding the soft tissue found in the trabeculae—contains very little fat [36], endogenous bone fat is not expected to impact our results. The muscle samples consisted of small remnants of meat adhering to the bone, which were cut off after the main muscle masses had been removed by the butcher.

Prior to the FA analysis, all tissues were manually triturated with a scalpel to obtain a homogenized product. The crude fat—which includes all types of lipids (triacylglycerols, phospholipids and cholesterol esters)—from the muscle and marrow samples was extracted using the method presented in Folch et al. [37], as modified by Dryer et al. [38] who recommended adding methanol in two separate aliquots in the initial steps of the procedure. Total FA (different lipid classes were not separated) in extracted lipids were then transesterified according to the method described by Chouinard et al. [39] using 100 μl of 0.5 *M* Na in methanol per ca 10 mg fat in 1 mL of hexane. Determination of the fatty acid profile was carried out with a gas chromatograph (HP 5890A Series II, Hewlett Packard, Palo Alto, CA) equipped with a 100-m CP-Sil 88 capillary column (Chrompack, Middelburg, the Netherlands) and a flame ionization detector, as described by Faucitano et al. [40]. The melting point of fat extracted from each sample was estimated as the weighted sum of the melting point of individual FA, as described by Toral et al. [41]. The comparisons that we performed include an examination of different indices, including the Δ^9^ desaturase index, the percentage of polyunsaturated FA (PUFA), the percentage of short chain saturated FA (Fig 3C) and the n-6/n-3 ratio. How these were calculated is presented in the accompanying figures and tables. For comparative purposes, we derived melting points from the caribou FA profiles published by Meng et al. [4]. Note that there are systematic differences between the two datasets likely because their earlier analyses could not identify certain categories of FA that can now be routinely identified (e.g., between *cis* and *trans* isomers, and between different PUFA), thanks to progress in gas chromatograph technology. The results that we present below begin with the limbs, as the FA composition of these body parts has previously been shown to be influenced by exposure to ambient temperature.

For comparison purposes, we calculated a number of percentages and ratios. The summed percentage of PUFA (%PUFA) includes the following FA: *cis*-9,12 18:2; *cis*-9,12,15 18:3; *cis*-6,9,12,15 18:4; *cis*-11,14 20:2; *cis*-8,11,14 20:3; *cis*-5,8,11,14 20:4; *cis*-5,8,11,14,17 20:5; *cis*-7,10,13,16 22:4; *cis*-4,7,10,13,16 22:5; *cis*-7,10,13,16,19 22:5; *cis*-4,7,10,13,16,19 22:6. The percentage of short chain saturated FA (%short chain saturated FA) focuses on the summed presence of 14:0 + 15:0. The following equation was used to derive the Δ^9^ desaturase index: (*cis*-9 14:1 + *cis*-9 16:1 + *cis*-9 18:1)/(14:0 + *cis*-9 14:1 + 16:0 + *cis*-9 16:1 + 18:0 + *cis*-9 18:1). This index was selected because it includes FA that are likely to have a significant impact on melting point. The n-6/n-3 ratio was calculated using this formula: n-6 PUFA/n-3 PUFA.

To assess change in FA composition, we measured distances between the sample sites and the body core. Due to the impracticality of taking these measurements in a commercial facility, an indirect approach was used. Distances between the sampling points and the trunk were measured in a diagram drawn to scale in Illustrator® with the proximal epiphysis of the femur and the proximal epiphysis of the humerus providing our “0” in the hindleg and foreleg, respectively. The measured (unitless) distances and the location of the sample sites are given in the S1 Fig.

### Statistical analysis

Variation in FA composition between different body parts was investigated using regression analyses. In addition to a visual inspection of the fit, the performance of the models was assessed by comparing the coefficients of determination, the distribution of the residuals and the Akaike information criterion (AICc), with the primacy being given to the latter in the comparisons [42]). The regression models were compared using the nonlinear fit function in *Past* v. 4.11 [43], whereas the residual analysis was performed in JMP ® v. 9 (SAS institute). Note that while the data points are generally shown separately for the two individuals, the best fit models were compared using the averaged data for the two individuals because there is good agreement in fatty acid variation between the animals. F ratio measures were also examined to determine whether the slope in linear models differed significantly from a null model [44].

## Results

**Fig 2:**
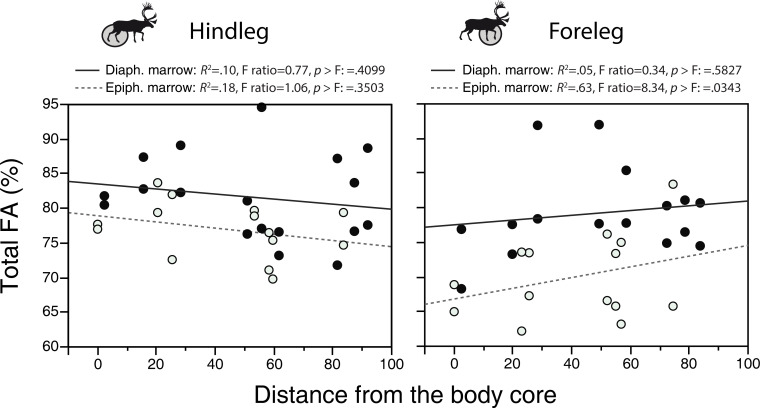
Relationship between the percentage of fatty acid (FA, % of crude fat) and sample distance in the diaphyseal and epiphyseal marrow of caribou. (distance data from S1 Fig; FA data from Table 1 and S1 File). Note that, with the exception of the slope for diaphyseal marrow in the foreleg, none of the slopes in this figure are significantly different from a null model. The regressions were calculated using the average for the two individuals. Closed circles: individual A; open circles: individual B.

**Fig 3:**
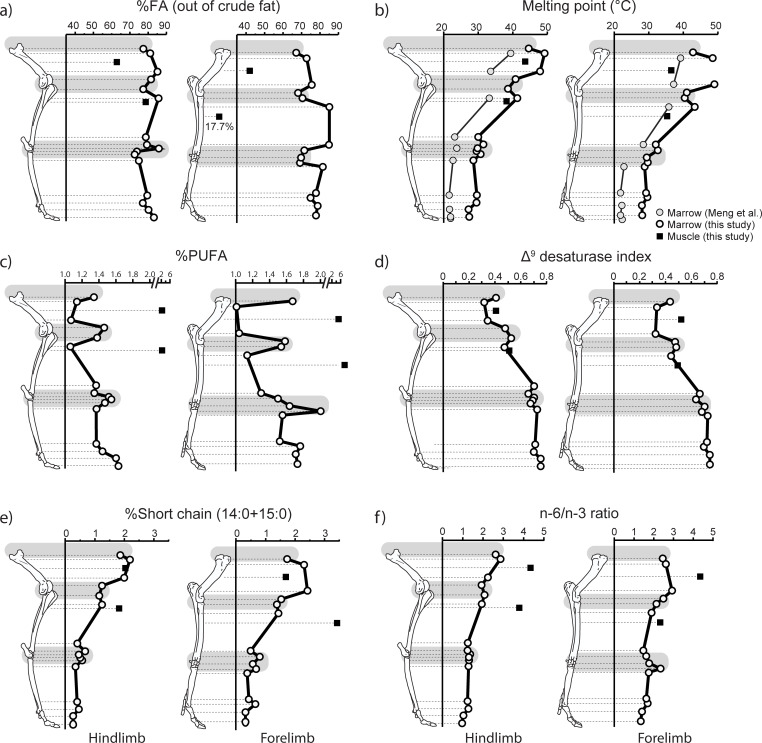
Fatty acid (FA) composition in the caribou limbs. a) percentage of FA (calculated out of crude fat); b) weighted melting point; c) percentage of polyunsaturated FA (PUFA); d) Δ^9^ desaturase index; e) %short chain saturated FA; f) n-6/n-3 FA ratio. Note the change of scale in c). The shaded areas correspond to areas of suspected significant hematopoiesis. The data points correspond to the mean for the two animals. The summed percentage of PUFA (%PUFA) includes these FA: *cis*-9,12 18:2; *cis*-9,12,15 18:3; *cis*-6,9,12,15 18:4; *cis*-11,14 20:2; *cis*-8,11,14 20:3; *cis*-5,8,11,14 20:4; *cis*-5,8,11,14,17 20:5; *cis*-7,10,13,16 22:4; *cis*-4,7,10,13,16 22:5; *cis*-7,10,13,16,19 22:5; *cis*-4,7,10,13,16,19 22:6. The percentage of short chain saturated FA (%short chain saturated FA) is the sum of 14:0 and 15:0. The Δ^9^ desaturase index was derived as follows: (*cis*-9 14:1 + *cis*-9 16:1 + *cis*-9 18:1)/(14:0 + *cis*-9 14:1 + 16:0 + *cis*-9 16:1 + 18:0 + *cis*-9 18:1). The n-6/n-3 ratio was obtained using this equation: n-6 PUFA/n-3 PUFA. Data from Tables 2, 3 and (S1 File). The comparative melting point data shown in b) were derived from Meng et al. [4]. The individual melting points used in the calculation of the weighted melting points (including those used in our conversion of Meng et al.’s data) are provided in the S1 File.

In the shaft cavity of the long bones, a locus normally dominated by cBMA in healthy (especially older) adults, the percentage of FA calculated on a crude fat basis is high (80.2 ± 4.5%, n = 12, Table 1). This observation contrasts with the lower percentages of FA in the epiphyseal portions of the same bones (73.8 ± 4.8%, n = 12, Table 1). These differences in percentages of FA between epiphyseal and diaphyseal marrow are particularly manifest when the averaged data are shown relative to sample distance (Fig 2) and when plotted on the caribou skeleton (Fig 3A). The lower values for the epiphyseal sites are likely due to an increased representation of membrane lipids, which is consistent with the known hematopoietic function of epiphyseal regions (they are normally dominated by rBMA in juveniles and younger adults). However, the epiphyseal regions of the distal metapodials (metatarsals and metacarpals) may represent an exception to this trend as they show only minor differences when compared to the adjacent diaphyseal marrow (Fig 3A). Moving to the trunk, values for the axial skeleton (75.4 ± 4.4%, n = 7, Table 1) are lower than for diaphyseal marrow sites and similar to epiphyseal marrow sites. We also note that the muscle tissues show low percentages of total lipids and FA, which suggests a low proportion of adipocytes in these tissues.

**Table 1:** Crude fat and fatty acid content of various body tissues in caribou. The values are averages for the two animals.

Anatomical site	Tissue (Reference #)	Crude fat^1^ (%, wet basis)	Total fatty acids (%, crude fat basis)	Total fatty acids^1^ (%, wet basis)
Mandible	Marrow (1)	60.1	78.0	46.8
Cervical vertebrae	Cancellous marrow (2)	28.0	70.9	19.9
	Meat (3)	5.5	69.8	3.9
Thoracic vertebrae	Cancellous marrow (4)	21.2	79.2	16.8
	Meat (5)	5.0	65.5	3.3
Lumbar vertebrae	Cancellous marrow (6)	23.2	76.5	17.8
	Meat (7)	7.3	64.3	4.7
Rib	Cancellous marrow (8)	17.2	75.9	13.0
	Meat (9)	9.7	84.1	7.5
Sternum	Cancellous marrow (10)	32.2	68.0	21.5
	Meat (11)	26.6	62.4	17.2
Scapula	Marrow (12)	51.2	79.8	40.7
	Cancellous marrow (13)	12.1	77.2	9.3
	Meat (14)	4.8	54.3	2.6
Humerus	Prox. epiphyseal marrow (15)	38.0	67.0	25.4
	Prox. diaphyseal marrow (16)	84.6	72.6	61.3
	Distal diaphyseal marrow (17)	75.1	75.5	56.8
	Distal epiphyseal marrow (18)	19.1	67.9	13.1
	Meat (19)	6.3	42.1	2.7
Radio-ulna	Prox. epiphyseal marrow (20)	14.5	70.4	10.6
	Prox. diaphyseal marrow (21)	75.4	85.2	64.0
	Distal diaphyseal marrow (22)	76.9	84.9	65.1
	Distal epiphyseal marrow (23)	15.6	71.4	11.2
	Meat (24)	11.3	17.7	2.0
Carpals	Cancellous marrow (25)	10.4	69.6	7.1
Metacarpal	Prox. epiphyseal marrow (26)	7.5	69.1	5.1
	Prox. diaphyseal marrow (27)	70.2	81.6	57.2
	Distal diaphyseal marrow (28)	64.5	77.6	50.1
	Distal epiphyseal marrow (29)	9.5	74.6	7.2
Anterior phalanx 1	Marrow (30)	75.1	78.8	59.2
Anterior phalanx 2	Marrow (31)	74.3	77.6	57.7
Pelvis	Marrow (32)	68.9	69.7	47.6
	Cancellous marrow (33)	24.1	80.0	19.4
	Meat (34)	4.2	56.1	2.3
Femur	Prox. epiphyseal marrow (35)	32.7	77.4	25.3
	Prox. diaphyseal marrow (36)	75.3	81.2	61.2
	Distal diaphyseal marrow (37)	74.3	85.1	63.1
	Distal epiphyseal marrow (38)	30.5	81.6	24.9
	Meat (39)	10.2	62.3	6.4
Tibia	Prox. epiphyseal marrow (40)	30.6	77.3	23.1
	Prox. diaphyseal marrow (41)	78.9	85.7	67.6
	Distal diaphyseal marrow (42)	72.2	78.7	56.8
	Distal epiphyseal marrow (43)	20.7	79.3	16.4
	Meat (44)	7.0	78.0	5.4
Calcaneus	Marrow (45)	55.7	85.9	46.5
Tarsals	Cancellous marrow (46)	7.7	73.8	5.7
Metatarsal	Prox. epiphyseal marrow (47)	12.7	72.6	9.2
	Prox. diaphyseal marrow (48)	79.0	74.9	59.1
	Distal diaphyseal marrow (49)	72.3	79.5	57.1
	Distal epiphyseal marrow (50)	15.9	77.1	12.2
Posterior phalanx 1	Marrow (51)	71.5	80.2	57.3
Posterior phalanx 2	Marrow (52)	63.5	83.2	52.8
Miscellaneous	Skin (53)	3.5	21.3	0.8
	Backfat (54)	89.8	74.7	66.8
	Tongue (55)	33.1	71.3	23.7
	Lungs and windpipe (56)	10.0	80.2	7.7
Mean and st. dev.	Cancellous marrow^1–2^	20.1 ±9.1	74.1 ±4.5	14.9 ±6.7
	axial skeleton only^1^	22.6 ±6.6	75.4 ±4.4	16.8 ±4.3
	epiphyseal regions only^1^	20.6 ±10.0	73.8 ±4.8	15.3 ±7.5
	Diaphyseal marrow^3^	74.9 ±5.0	80.2 ±4.5	59.9 ±4.8
	Meat	10.9 ±9.2	60.7 ±17.4	6.8 ±6.7

^1^The low values for crude fat and Total fat weight (%, wet basis) for the cancellous marrow are, in part, due to the presence of bone fragments in the samples.

^2^Cancellous marrow includes the following: vertebrae, ribs, sternum, pelvis, scapulae, epiphyseal regions of long bones, as well as carpals and tarsals. The axial skeleton includes all of these bones to the exclusion of the limb bones.

^3^Diaphyseal marrow includes all of the shaft portions of the long bones. Although not “true” long bones, metapodials are treated here as long bones due to their large marrow cavity.

The gas chromatography analysis allowed the identification and quantification of 33 different FA varying from 14 to 22 carbon chain lengths (Table 2). In the appendicular skeleton, we note a gradual increase in the proportion of the *cis*-9 monounsaturated FA as one progresses away from the body core (Table 2). This increase is primarily expressed in the form of a greater proportion of oleic acid (*cis*-9 18:1) and, to a lesser extent, palmitoleic acid (*cis*-9 16:1) in the extremities. Conversely, a gradual decrease in several classes of saturated FA—mostly palmitic acid (16:0) and stearic acid (18:0)—is observed distally. These changes in the FA composition of appendicular marrow produce a steady decrease in average fat melting point as one moves toward the extremities (Fig 3B). However, the average melting point of the epiphyseal regions is consistently lower than predicted by the FA pattern for diaphyseal marrow (Fig 3B, Table 2). These lower melting points may signal an increased presence of lipids from membranes in epiphyseal regions, which is consistent with the higher percentages of PUFA observed in the same regions (Fig 3C). When compared to the shaft regions and excluding the metapodials, the epiphyseal regions also show higher values for the Δ^9^ desaturase index (Fig 3D) and lower percentages of short chain saturated FA (Fig 3E). In contrast, changes in the n-3/n-6 ratio (Table 3) are small, with a possible pattern of decrease as one moves distally (Fig 3F).

**Table 2:** Fatty acid profile and calculated melting point of lipid extracted from varying body tissues in caribou. The values are averages for the two animals.

Anatomical		Fatty acid (% by weight)								Δ^9^ desatur.	Melting
site	Tissue (Reference #)	14:0	16:0	*cis*-9 16:1	18:0	*cis*-9 18:1	*cis*-11 18:1	*cis*-9,12 18:2	Others^1^	Index^2^	Point (°C)
Mandible	Marrow (1)	1.6	33.9	0.9	27.2	29.4	1.0	0.5	5.5	0.33	48.6
Cervical vert.	Cancellous marrow (2)	1.7	31.8	0.9	26.7	30.4	1.2	1.1	6.3	0.34	47.1
	Meat (3)	1.4	30.0	0.9	25.9	33.3	1.4	1.7	5.4	0.37	45.1
Thoracic vert.	Cancellous marrow (4)	1.9	33.7	1.0	27.0	29.1	1.1	1.1	5.0	0.33	48.2
	Meat (5)	1.3	29.1	1.0	26.9	30.7	1.5	3.7	5.7	0.36	43.5
Lumbar vert.	Cancellous marrow (6)	1.6	31.2	1.1	26.9	32.6	1.1	0.8	4.8	0.36	46.7
	Meat (7)	1.4	29.4	0.9	28.2	32.7	1.4	1.4	4.6	0.36	46.0
Ribs	Cancellous marrow (8)	2.3	33.0	0.9	29.9	27.5	1.0	0.9	4.5	0.30	49.5
	Meat (9)	1.7	30.0	0.9	28.8	30.6	1.1	1.5	5.3	0.34	46.7
Sternum	Cancellous marrow (10)	2.3	33.2	1.1	27.0	29.1	1.1	1.1	5.2	0.33	48.0
	Meat (11)	1.7	30.8	1.6	27.5	32.1	1.0	1.2	4.1	0.36	46.4
Scapula	Marrow (12)	2.6	37.7	1.1	27.0	24.5	1.0	0.9	5.2	0.28	50.6
	Cancellous marrow (13)	2.5	34.7	1.1	27.0	27.4	1.1	1.0	5.4	0.31	49.0
	Meat (14)	1.4	26.1	1.2	19.4	42.6	1.5	2.4	5.5	0.48	39.3
Humerus	Prox. epiphyseal marrow (15)	1.4	28.9	1.3	21.3	38.6	1.4	1.0	6.3	0.44	42.8
	Prox. diaphyseal marrow (16)	1.9	32.0	0.9	27.8	29.5	1.1	0.6	6.3	0.33	48.5
	Distal diaphyseal marrow (17)	2.0	32.2	0.9	28.6	28.6	1.2	0.6	6.0	0.32	49.0
	Distal epiphyseal marrow (18)	1.2	27.2	1.5	19.8	41.5	1.8	0.9	6.1	0.47	41.0
	Meat (19)	1.2	24.3	1.4	17.3	44.6	1.8	2.8	6.6	0.52	36.5
Radio-ulna	Prox. epiphyseal marrow (20)	1.1	28.1	1.7	18.6	42.6	1.8	0.8	5.3	0.48	40.4
	Prox. diaphyseal marrow (21)	1.2	26.5	1.1	23.9	39.1	1.5	0.6	6.2	0.44	43.3
	Distal diaphyseal marrow (22)	0.4	17.6	2.1	12.4	57.3	3.5	0.6	6.2	0.66	32.0
	Distal epiphyseal marrow (23)	0.6	21.7	3.0	10.6	54.2	3.5	0.7	5.6	0.64	32.6
	Meat (24)	2.8	25.3	1.6	15.5	39.8	1.4	3.2	10.5	0.49	35.3
Carpals	Cancellous marrow (25)	0.4	17.1	2.9	9.0	60.1	4.0	0.9	5.8	0.70	29.4
Metacarpal	Prox. epiphyseal marrow (26)	0.5	20.1	4.1	8.0	56.0	4.6	0.8	5.9	0.68	29.7
	Prox. diaphyseal marrow (27)	0.2	14.5	2.4	9.3	61.8	4.0	0.8	6.9	0.73	28.8
	Distal diaphyseal marrow (28)	0.3	16.0	2.5	9.0	62.0	3.2	0.8	6.2	0.72	29.2
	Distal epiphyseal marrow (29)	0.4	19.0	3.1	8.3	58.8	4.2	0.8	5.4	0.69	29.6
Ant. phalanx 1	Marrow (30)	0.2	14.7	2.3	8.0	63.0	4.3	0.8	6.8	0.74	28.0
Ant. phalanx 2	Marrow (31)	0.2	14.5	2.3	8.4	63.2	4.0	0.8	6.6	0.74	28.1
Pelvis	Marrow (32)	2.3	35.5	1.1	27.7	26.2	1.1	0.9	5.3	0.29	49.9
	Cancellous marrow (33)	1.7	29.5	1.0	23.4	37.6	1.0	0.8	4.9	0.42	44.0
	Meat (34)	1.1	24.2	1.1	18.9	44.1	1.6	3.3	5.7	0.51	37.3
Femur	Prox. epiphyseal marrow (35)	1.5	30.1	1.2	23.5	36.2	1.2	0.8	5.5	0.40	44.6
	Prox. diaphyseal marrow (36)	1.8	32.0	0.8	29.4	28.4	0.9	0.7	5.9	0.32	49.2
	Distal diaphyseal marrow (37)	1.6	30.9	0.8	28.2	30.8	1.1	0.6	6.0	0.34	47.9
	Distal epiphyseal marrow (38)	1.0	26.9	1.3	19.8	42.3	2.0	0.8	5.9	0.48	40.8
	Meat (39)	1.7	30.5	1.2	22.3	36.2	1.4	1.5	5.3	0.41	43.6
Tibia	Prox. epiphyseal marrow (40)	0.9	25.0	1.4	17.8	46.9	1.8	0.7	5.4	0.53	38.6
	Prox. diaphyseal marrow (41)	1.0	27.1	1.5	20.2	41.8	1.9	0.6	5.9	0.47	41.3
	Distal diaphyseal marrow (42)	0.3	16.1	2.3	10.1	60.2	4.0	0.7	6.4	0.70	30.0
	Distal epiphyseal marrow (43)	0.6	21.7	3.3	9.1	55.9	4.0	0.6	4.7	0.65	31.5
	Meat (44)	1.6	27.8	1.7	16.1	45.8	1.5	1.3	4.3	0.51	38.2
Calcaneus	Marrow (45)	0.3	16.9	2.8	9.4	59.6	4.0	0.7	6.2	0.70	29.8
Tarsals	Cancellous marrow (46)	0.4	19.7	3.2	7.7	59.3	4.4	0.7	4.7	0.69	29.5
Metatarsal	Prox. epiphyseal marrow (47)	0.5	20.6	3.0	8.6	57.4	4.3	0.7	4.9	0.67	30.7
	Prox. diaphyseal marrow (48)	0.2	15.6	2.4	8.4	61.1	5.7	0.6	5.8	0.72	28.6
	Distal diaphyseal marrow (49)	0.3	17.1	2.4	8.8	60.7	4.0	0.6	6.1	0.71	29.6
	Distal epiphyseal marrow (50)	0.4	19.3	3.0	7.6	59.7	4.4	0.6	5.0	0.70	29.5
Post. phalanx 1	Marrow (51)	0.2	14.6	2.6	7.0	63.9	4.8	0.7	6.2	0.75	27.2
Post. phalanx 2	Marrow (52)	0.2	14.6	2.1	7.3	64.6	4.5	0.7	6.2	0.75	27.4
Miscellaneous	Skin (53)	1.6	24.4	0.9	23.2	30.6	0.6	1.5	17.6	0.39	40.8
	Backfat (54)	3.4	33.9	1.5	26.9	28.8	0.9	0.4	4.2	0.32	47.9
	Tongue (55)	2.1	35.1	1.3	22.4	31.8	1.5	0.9	4.8	0.36	46.2
	Lungs and windpipe (56)	1.2	29.1	1.4	21.1	40.2	1.7	0.9	4.4	0.45	42.0

^1^See (S1 File) for complete profiles

^2^(*cis*-9 14:1 + *cis*-9 16:1 + *cis*-9 18:1) / (14:0 + *cis*-9 14:1 + 16:0 + *cis*-9 16:1 + 18:0 + *cis*-9 18:1)

**Table 3:** Polyunsaturated fatty acid family in lipids extracted from various body tissues in caribou.

		Fatty acid (% by weight)			
Anatomical site	Tissue (Reference #)	Total n-6^1^	Total n-3^1^	Sum n-6+n-3	Ratio n-6/n-3
Mandible	Marrow (1)	0.75	0.33	1.08	2.30
Cervical vertebrae	Cancellous marrow (2)	1.51	0.56	2.07	2.72
	Meat (3)	2.29	0.45	2.74	5.04
Thoracic vertebrae	Cancellous marrow (4)	1.27	0.33	1.61	3.82
	Meat (5)	5.09	1.04	6.13	4.91
Lumbar vertebrae	Cancellous marrow (6)	0.96	0.34	1.30	2.83
	Meat (7)	1.85	0.45	2.30	4.10
Rib	Cancellous marrow (8)	1.12	0.29	1.41	3.84
	Meat (9)	2.12	0.56	2.67	3.81
Sternum	Cancellous marrow (10)	1.33	0.37	1.70	3.58
	Meat (11)	1.48	0.38	1.86	3.85
Scapula	Marrow (12)	1.02	0.21	1.23	4.98
	Cancellous marrow (13)	1.20	0.26	1.47	4.55
	Meat (14)	3.11	0.54	3.65	5.73
Humerus	Prox. epiphyseal marrow (15)	1.18	0.49	1.67	2.40
	Prox. diaphyseal marrow (16)	0.72	0.29	1.01	2.52
	Distal diaphyseal marrow (17)	0.77	0.27	1.04	2.79
	Distal epiphyseal marrow (18)	1.12	0.47	1.59	2.41
	Meat (19)	4.09	0.97	5.06	4.20
Radio-ulna	Prox. epiphyseal marrow (20)	1.04	0.49	1.54	2.11
	Prox. diaphyseal marrow (21)	0.74	0.39	1.13	1.88
	Distal diaphyseal marrow (22)	0.77	0.53	1.30	1.47
	Distal epiphyseal marrow (23)	0.92	0.58	1.50	1.60
	Meat (24)	5.53	2.31	7.84	2.39
Carpals	Cancellous marrow (25)	1.04	0.60	1.64	1.73
Metacarpal	Prox. epiphyseal marrow (26)	1.40	0.61	2.01	2.28
	Prox. diaphyseal marrow (27)	0.97	0.58	1.55	1.67
	Distal diaphyseal marrow (28)	0.92	0.60	1.52	1.55
	Distal epiphyseal marrow (29)	1.10	0.67	1.77	1.65
Anterior phalanx 1	Marrow (30)	0.96	0.75	1.71	1.29
Anterior phalanx 2	Marrow (31)	0.96	0.77	1.73	1.26
Pelvis	Marrow (32)	1.01	0.26	1.27	3.83
	Cancellous marrow (33)	1.10	0.41	1.51	2.65
	Meat (34)	4.41	0.80	5.21	5.53
Femur	Prox. epiphyseal marrow (35)	0.97	0.37	1.34	2.62
	Prox. diaphyseal marrow (36)	0.84	0.29	1.14	2.88
	Distal diaphyseal marrow (37)	0.74	0.33	1.07	2.23
	Distal epiphyseal marrow (38)	0.97	0.50	1.46	1.93
	Meat (39)	1.91	0.47	2.38	4.08
Tibia	Prox. epiphyseal marrow (40)	0.92	0.45	1.37	2.06
	Prox. diaphyseal marrow (41)	0.70	0.36	1.06	1.92
	Distal diaphyseal marrow (42)	0.76	0.61	1.37	1.24
	Distal epiphyseal marrow (43)	0.74	0.61	1.34	1.22
	Meat (44)	1.91	0.50	2.41	3.84
Calcaneus	Marrow (45)	0.87	0.65	1.51	1.34
Tarsals	Cancellous marrow (46)	0.87	0.67	1.55	1.30
Metatarsal	Prox. epiphyseal marrow (47)	0.83	0.63	1.46	1.31
	Prox. diaphyseal marrow (48)	0.77	0.60	1.37	1.28
	Distal diaphyseal marrow (49)	0.76	0.61	1.37	1.24
	Distal epiphyseal marrow (50)	0.81	0.63	1.44	1.28
Posterior phalanx 1	Marrow (51)	0.81	0.79	1.60	1.02
Posterior phalanx 2	Marrow (52)	0.80	0.82	1.63	0.98
Miscellaneous	Skin (53)	4.13	1.87	6.00	2.20
	Backfat (54)	0.54	0.30	0.84	1.82
	Tongue (55)	1.12	0.38	1.51	2.91
	Lungs and windpipe (56)	1.19	0.51	1.70	2.32

^1^See (S1 File) for complete profiles.

**Fig 4:**
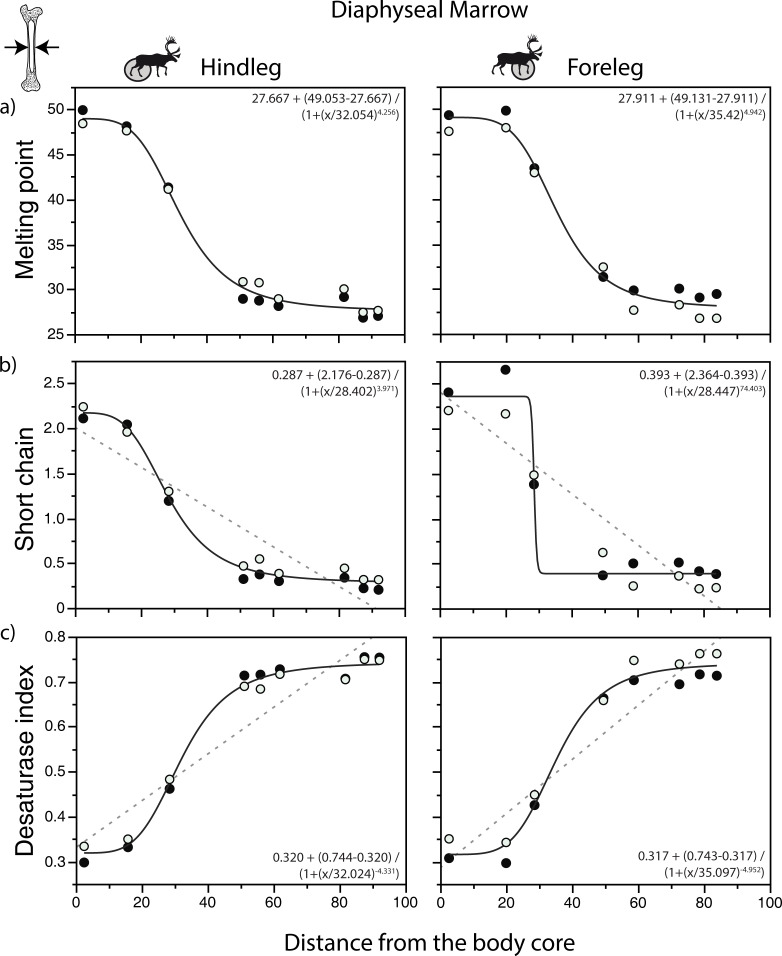
Relationship between fatty acid (FA) parameters and sample distance in the diaphyseal marrow of caribou. a) weighted melting point (in °C); b) percentage of short chain saturated FA; c) Δ^9^ desaturase index. Bold curves assume a sigmoidal function; dashed curves are relationships that show a better fit with a linear function according to the AICc values (S1 Table). Melting point and FA values calculated as in Fig 3. Distance data from S1 Fig, FA data from Table 2. The regressions were calculated using the mean for the two individuals. Closed circles: individual A; open circles: individual B.

**Fig 5:**
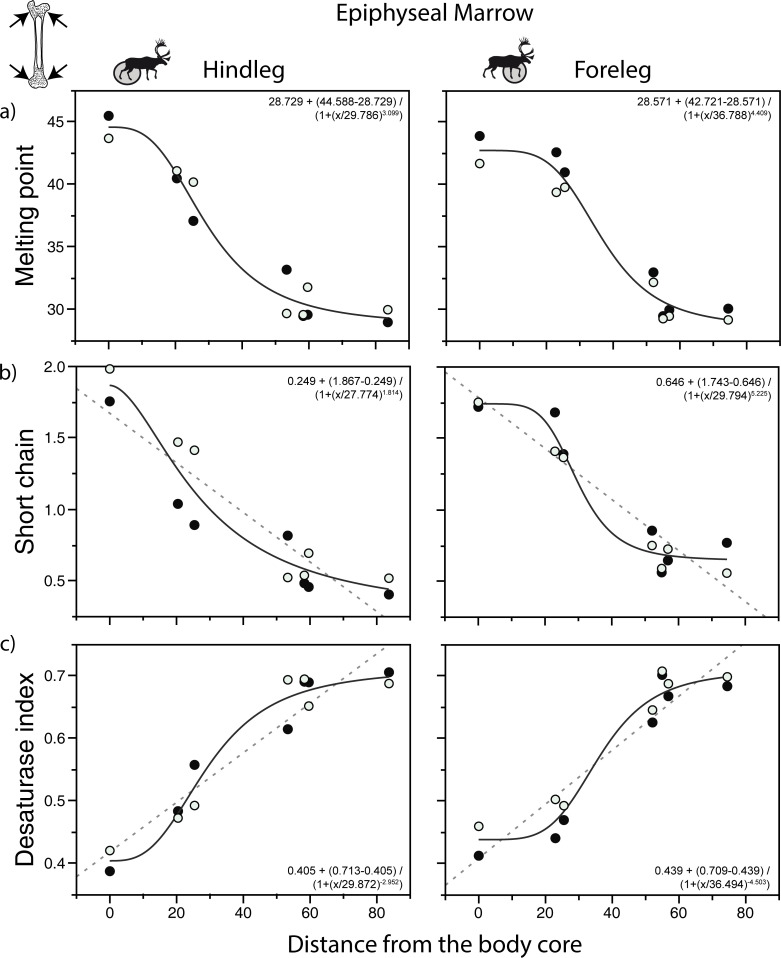
Relationship between fatty acid (FA) parameters and sample distance in the epiphyseal marrow of caribou. a) weighted melting point (in °C); b) percentage of short chain saturated FA; c) Δ^9^ desaturase index. Bold curves assume a sigmoidal function; dashed curves are relationships that show a better fit with a linear function according to the AICc values (S1 Table). Melting point and FA values calculated as in Fig 3. Distance data from S1 Fig, FA data from Table 2. The regressions were calculated using the mean for the two individuals. Closed circles: individual A; open circles: individual B. “0” were replaced by “0.0001” in the calculation of the sigmoidal function as the model cannot be computed with the former values.

Fig 4 focuses on several key measures of FA variation in the legs. In diaphyseal marrow, the AICc values (S1 Table) indicate that a sigmoidal (Hill) function most parsimoniously describes the relationship between sample distance and the weighted melting point (Fig 4A). In contrast, according to the AICc values, a linear model provides a better fit when the percentage of short chain saturated FA or the Δ^9^ desaturase index are the response variables (S1 Table). However, because a visual inspection of the plots suggest a good fit with a sigmoidal function and because the accuracy of the AICc values is likely limited by the small numbers of data points, the relationships for the percentage of short chain saturated FA and the Δ^9^ desaturase index are shown using both linear (dashed lines) and sigmoidal (bold lines) functions in Fig 4B, 4C. The best fit models and the patterns of change are similar in epiphyseal marrow (Fig 5, S1 Table). An analysis of residuals (S2, S3 Figs) and normal quantile plots (S4, S5 Figs) is consistent with these interpretations.

**Fig 6:**
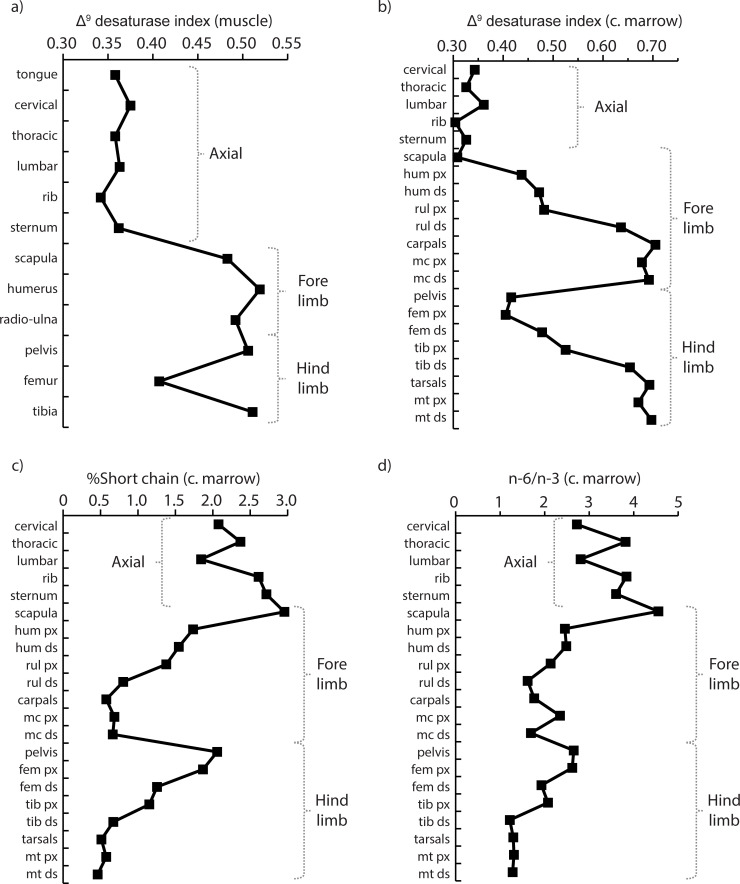
Fatty acids (FA) in the axial and appendicular skeleton of caribou. a) Δ^9^ desaturase index in muscle tissues; b) Δ^9^ desaturase index in cancellous marrow; c) percentage of short chain saturated FA in cancellous marrow; d) n-6/n-3 ratio in cancellous marrow. Comparisons for marrow exclude the shaft cavities. The data points correspond to the mean for the two animals. Data from Tables 2, 3 and (S1 File). Abbreviations: c. marrow = cancellous marrow; hum = humerus; rul = radio-ulna; mc = metacarpal; fem = femur; tib = tibia; mt = metatarsal; px = proximal epiphysis; ds = distal epiphysis.

A comparison of the limbs with the axial skeleton shows several interesting patterns. In muscle tissues, values for the Δ^9^ desaturase index are systematically lower in the body core (tongue to sternum: 0.360 ± .011, n = 6) than the limbs (scapula, pelvis and long bones: 0.486 ± .041, n = 6, Fig 6A), a trend also observed in cancellous marrow (cervical to sternum: 0.332 ± .021, n = 5; scapula, pelvis and limb bones: 0.559 ± .132, n = 16, Fig 6B). We note that the percentage of short chain saturated FA (Fig 6C) and the n-6/n-3 ratio (Fig 6D) are higher in the cancellous marrow of the axial skeleton than that of the limbs where a steady decrease is observed as one proceeds distally.

## Discussion

Previous research on the thermal adaptation of limbs in cold-adapted mammals has largely focused on FA variation in the cBMA of long bones with occasional comparisons with other types of soft tissues [4, 24, 29]. In the present study, the FA profiles of a wide range of lipid-containing tissues in caribou were compared between and within different anatomical locations. Most of the bone marrow and muscle samples that we examined varied in terms of lipid proportions. The FA profiles suggest that the diaphyseal marrow and backfat are dominated by triacylglycerols derived from adipocytes (loosely connected with collagen) whereas the other tissues—such as muscles, and perhaps, rBMA—appear to show greater proportions of phospholipids and cholesterol esters derived from cell membranes. The latter is supported by: i) lower proportions of total FA expressed in percentage of extracted crude fat, and ii) greater concentrations of total n-6 and n-3 PUFA in epiphyseal as compared with diaphyseal marrow. However, additional, more fine-grained analyses will be needed to validate these conclusions.

Our analysis reveals other important differences between the epiphyseal and diaphyseal portions of the long bones, the former regions showing higher values for the Δ^9^ desaturase index and lower melting points. In the caribou females that we sampled, both scored as prime adults, the epiphyseal regions of the long bones were apparently actively involved in hematopoiesis, as suggested by the greater concentrations of total n-6 and n-3 PUFA observed in the extremities of long bones. Vanhie et al. [45] reported that PUFA of the n-6 and n-3 families may have distinct effects on hematopoiesis in studies conducted in vitro. Moreover, n-3 PUFA appear to decrease inflammation and provide substrates for β-oxidation, which is known to play an important role in hematopoiesis regulation [45]. If confirmed under more controlled experimental conditions, this finding would be consistent with observations made in humans and many other mammals [15].

In agreement with Meng et al.’s [4] observations about the FA composition of cBMA, the diaphyseal regions of the long bones show a pattern of desaturation toward the extremities, which contributes to lowering the melting point of the adipose tissues. A similar trend is seen in the epiphyseal regions. The distal decrease in melting point in diaphyseal and epiphyseal regions supports the hypothesis of a physiological adaptation of the lipid component of cells to the marked heterothermia that may be displayed in reindeer legs [2, 34]. This is because a low melting point allows for soft tissues of the peripheral parts—including extremities that are less insulated and are allowed to cool more than the trunk in order to limit heat loss rate—to remain supple when facing cool thermal conditions.

Our regression analyses suggest that the relationships between FA composition and sample distance fit well with a sigmoidal function, with most of the changes occurring in the zeugopodium (tibia and radio-ulna). However, this interpretation must be viewed with caution given the small number of data available for these skeletal elements. Bones that are found near the body core (e.g., femur and humerus) and close to the ground (metapodials, carpals, tarsals and phalanges) vary little as they are similarly dominated by the same range of unsaturated FA. The improved fit with a sigmoidal function seen in the weighted melting point may reflect the fact that it includes the complete FA profile—and for this reason, probably provides a more robust signal—in comparison to the small range of FA included in the percentage of short chain saturated FA and the Δ^9^ desaturase index. Additional studies will be needed to determine whether these trends can be generalized to other species as well.

It is known that FA with a higher degree of unsaturation—especially those high in PUFA with their double bonds located near the methyl end—can, when need arises, be mobilized more readily than saturated FA [46–49]. For instance, in situations of impaired energy balance, the proportion of unsaturated and long-chain FA decreases, presumably because they are preferentially mobilized from triacylglycerols of adipose tissues [50–52], including bone marrow fat (presumably compensated by an increase in water content [24]), and brown adipose tissue [53]. However, additional work will be needed to assess whether there are differences in the fat mobilization process between rBMA and cBMA and whether processes of fat mobilization occur faster in the foreleg and neck than in other parts as claimed by Nunamiut (Inuit) caribou hunters of Alaska [54].

Previous studies that compared different muscles in terrestrial mammals have often stressed the lack of FA variation in intramuscular fat between muscles of single animals [32, 55] and in patterns of FA desaturation between species living at different latitudes [56]. Our results show relatively minor changes in FA composition between different muscles in the axial skeleton. However, the Δ^9^ desaturase index shows clear and apparently systematic differences in the muscles between the axial and appendicular skeleton. The pattern that we uncovered is consistent with appendicular muscle tissues being more unsaturated than those of the axial skeleton [28, 57, 58], presumably as an adaptation to the cooler temperature seen in the limbs of mammals due to their thermoregulatory vasoconstrictor responses aimed at minimizing heat loss in the limbs. As a last point, it is well established that PUFA are critical in influencing the fluidity, permeability and protein binding functions of cell membranes. The changes in PUFA abundance seen in the bone marrow and muscle tissues that we examined are in agreement with this interpretation.

## Conclusion

It is increasingly clear that the rBMA in the epiphyseal regions differ morphologically and functionally from that found in the cBMA of diaphyseal regions [22, 23]. Assuming that our interpretation of BMA distribution in the limb samples is correct, our analysis suggests that cBMA and rBMA vary systematically in terms of FA composition, with both subtypes of adipocytes showing a distal increase in the degree of unsaturation and an overall decrease in fat melting point in the limbs. While variation in FA composition seems limited in muscles of the body core, the cell membranes of muscle tissues show patterns of change of FA in the limbs, including an increase in the Δ^9^ desaturase index, that are potentially consistent with an adaptation to exposure to ambient temperature. Given that patterns of thermoregulation seem widely shared among terrestrial mammals—with frequent contrasts being seen between the warmer core and the more heterothermic extremities and appendages—we suggest that the trends of FA composition that we observed in the appendicular skeleton of caribou is also characteristic of other species, possibly including humans.

## Supporting information

S1 File Complete FA profiles for the two caribou individuals. (XLSX)

S1 Table Akaike information criterion (AICc) values for the relationship between sample distance and the FA data. (DOCX)

S1 Fig Measured distances between the sample sites in the limbs and the body core in the virtual skeleton of a caribou. (DOCX)

S2 Fig Plots of residuals for the relationship between FA composition and sample distance in the diaphyseal marrow of caribou (linear model). (DOCX)

S3 Fig Plots of residuals for the relationship between FA composition and sample distance in the epiphyseal marrow of caribou (linear model). (DOCX)

S4 Fig Normal quantile plots for the relationship between FA composition and sample distance in the diaphyseal marrow of caribou (linear model). (DOCX)

S5 Fig Normal quantile plots for the relationship between FA composition and sample distance in the epiphyseal marrow of caribou (linear model). (DOCX)
