# Supplementary material for: Thermal adaptation and fatty acid profiles of bone marrow and muscles in mammals: Implications of a study of caribou (Rangifer tarandus caribou)
Source: PLoS One. 2022 Dec 1;17(12):e0268593. doi: 10.1371/journal.pone.0268593 (PMC9714762; doi:10.1371/journal.pone.0268593)
Supplement: S1 Table — (DOCX) [file pone.0268593.s002.docx]

|  |  | Hindleg | | | Foreleg | | |
| --- | --- | --- | --- | --- | --- | --- | --- |
| Diaphyseal marrow | | Linear | Quadratic | Sigmoidal | Linear | Quadratic | Sigmoidal |
|  | Melting point | 94.623 | 41.639 | 21.538 | 68.919 | 65.899 | 24.519 |
|  | Short chain FA | 6.7583 | 10.97 | 18.016 | 7.2519 | 12.617 | 21.365 |
|  | Δ^9^ desaturase index | 6.0353 | 10.813 | 18.001 | 6.4254 | 12.022 | 21.335 |
|  |  |  |  |  |  |  |  |
| Epiphyseal marrow | |  |  |  |  |  |  |
|  | Melting point | 25.241 | 21.959 | 29.38 | 30.079 | 37.078 | 32.489 |
|  | Short chain FA | 7.1386 | 14.006 | 28.009 | 7.1389 | 14.129 | 28.024 |
|  | Δ^9^ desaturase index | 7.0062 | 14.003 | 28.001 | 7.009 | 14.009 | 28.002 |

Table S1. Akaike information criterion (AICc) values for the relationship between sample distance and the FA parameters. Best fit models are identified by gray shading. For consistency, although the best fit model for melting point in the hindleg is a quadratic model, the data are shown according to a sigmoidal function in Fig 5. Melting point and FA values calculated as in Fig. 3. Distance data from Fig. S1, FA data from Table 2. “0” were replaced “0.0001” in the calculation of the sigmoidal function as the model cannot be computed with the former values.
