# Supplementary material for: Thermal adaptation and fatty acid profiles of bone marrow and muscles in mammals: Implications of a study of caribou (Rangifer tarandus caribou)
Source: PLoS One. 2022 Dec 1;17(12):e0268593. doi: 10.1371/journal.pone.0268593 (PMC9714762; doi:10.1371/journal.pone.0268593)
Supplement: S1 Fig — (DOCX) [file pone.0268593.s003.docx]

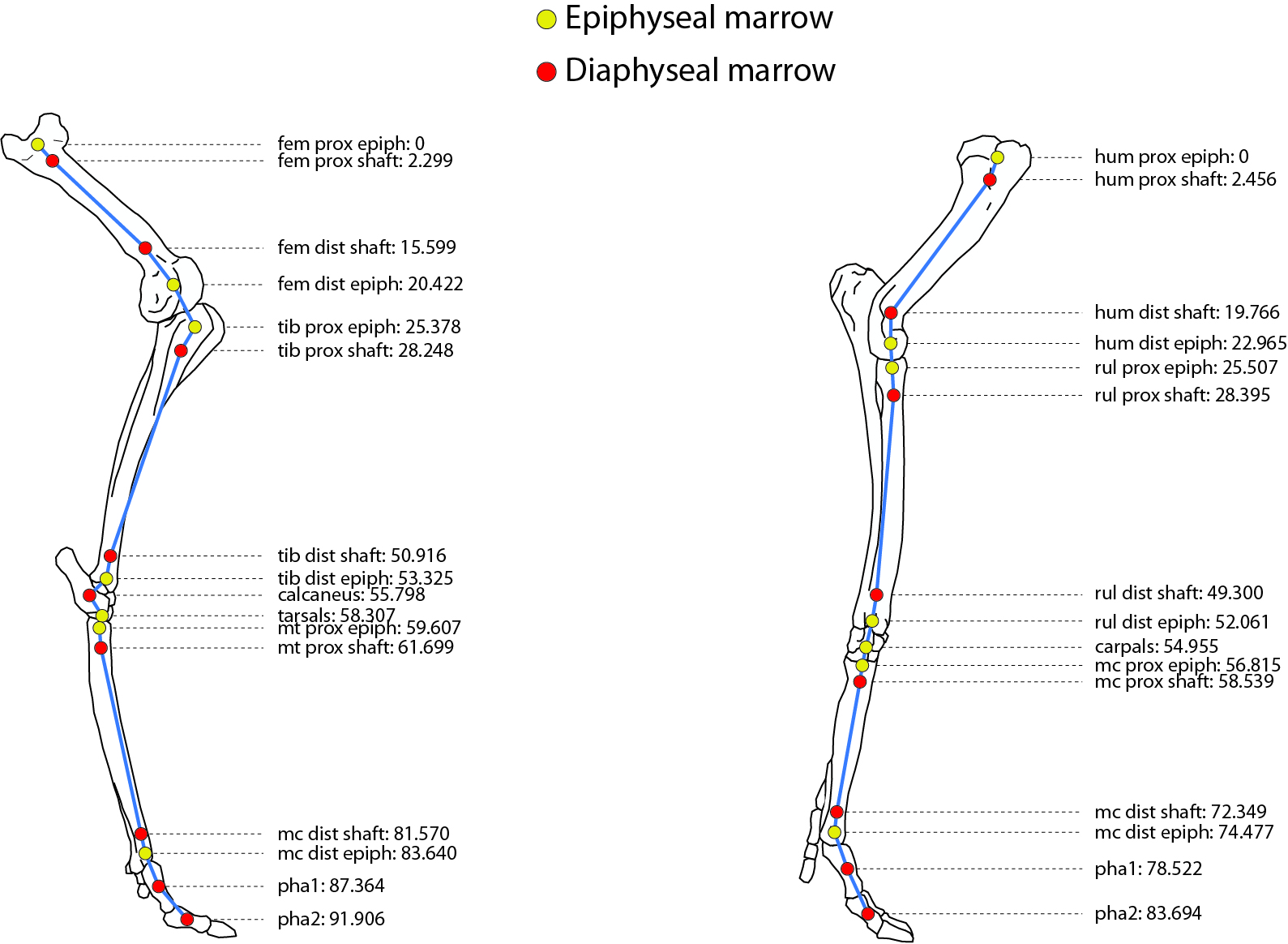


**Fig. S1. Measured distances between the sample sites in the limbs and the body core in the virtual skeleton of a caribou.** The (unitless) distances were derived in Illustrator® using the sample sites and blue lines shown in the illustration.
