# Supplementary material for: Thermal adaptation and fatty acid profiles of bone marrow and muscles in mammals: Implications of a study of caribou (Rangifer tarandus caribou)
Source: PLoS One. 2022 Dec 1;17(12):e0268593. doi: 10.1371/journal.pone.0268593 (PMC9714762; doi:10.1371/journal.pone.0268593)
Supplement: S4 Fig — (DOCX) [file pone.0268593.s006.docx]

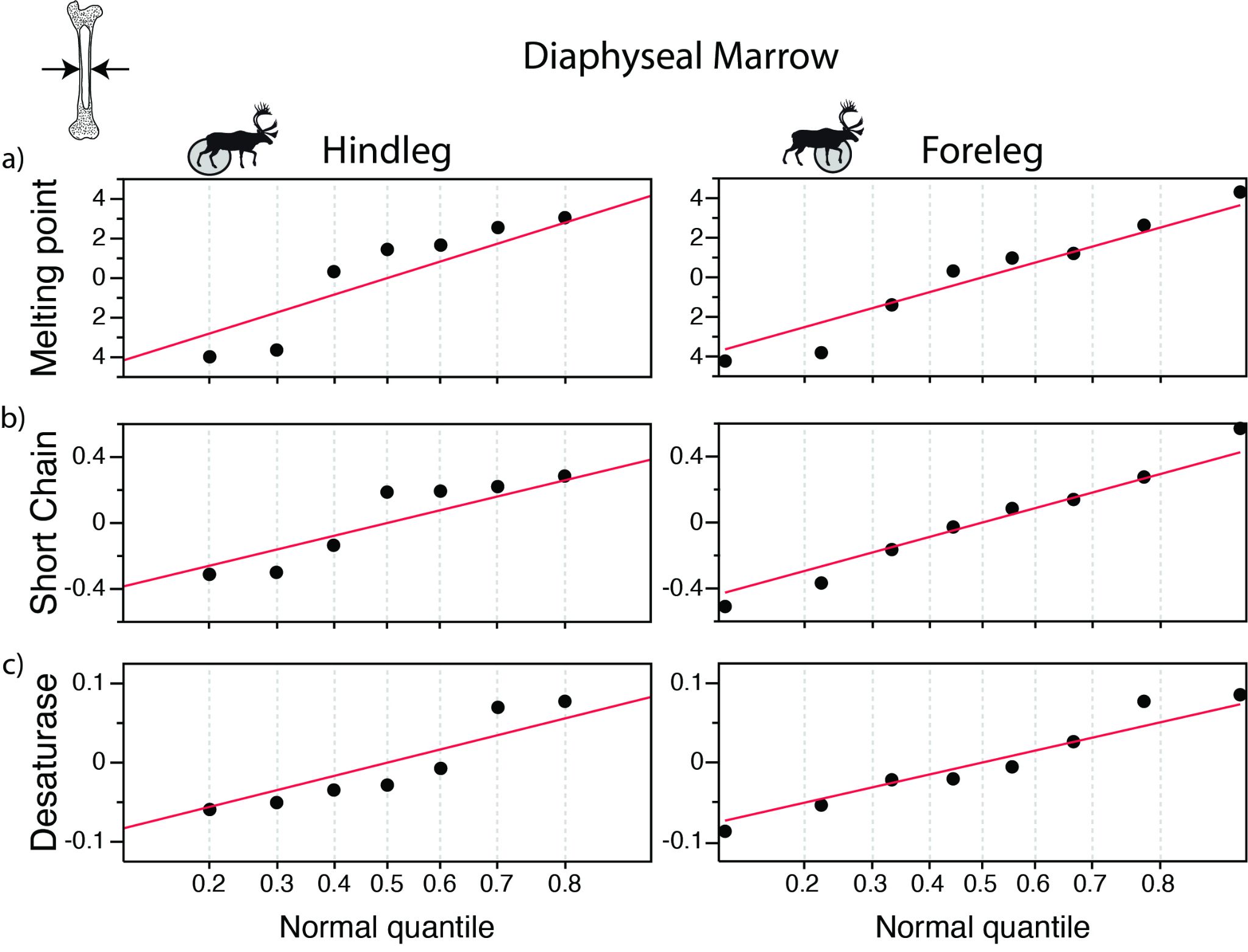


**Fig S4. Normal quantile plots for the relationship between fatty acid (FA) parameters and sample distance in the diaphyseal marrow of caribou (linear model)**: a) weighted melting point (in °C); b) percentage of short chain saturated FA; c) Δ^9^ desaturase index. Melting point and FA values calculated as in Fig. 3. The plots correspond to the average values for the two individuals. Distance data from Fig. S1, melting point and FA data from Table 2.
